# iHofman: a predictive model integrating high-order and low-order features with weighted attention mechanisms for circRNA-miRNA interactions

**DOI:** 10.1186/s12915-025-02260-5

**Published:** 2025-06-09

**Authors:** Chang-Qing Yu, Chen Jiang, Lei Wang, Zhu-Hong You, Xin-Fei Wang, Meng-Meng Wei, Tai-Long Shi, Si-Zhe Liang

**Affiliations:** 1 School of Information Engineering, Xijing Univerity, Xi’an, 710123 China; 2 Guangxi Key Lab of Human-Machine Interaction and Intelligent Decision, Guangxi Academy of Science, Nanning, 530007 China; 3 School of Computer Science and Technology, China University of Mining and Technology, Xuzhou, 221116 China; 4 School of Computer Science, Northwestern Polytechnical University, Xi’an, 710129 China; 5 College of Computer Science and Technology, Jilin University, Changchun, 130012 China

**Keywords:** CircRNA, MiRNA, CircRNA-miRNA interaction, FastText, GraRep, Weighted attention mechanisms

## Abstract

**Background:**

Increasing research indicates that the complex interactions between circular RNAs (circRNAs) and microRNAs (miRNAs) are critical for diagnosing and treating various human diseases. Consequently, accurately predicting potential circRNA-miRNA interactions (CMIs) has become increasingly important and urgent. Traditional biological experiments, however, are often labor-intensive, time-consuming, and prone to external influences.

**Results:**

To tackle this challenge, we present a novel model, iHofman, designed to predict CMIs by *i*ntegrating *h*igh-order and l*o*w-order *f*eatures with weighted attention *m*ech*an*isms. Specifically, we first extract sequence and structural information representations using FastText and GraRep, respectively, and capture high-order and low-order features from sequence information representations using stacked autoencoders. Subsequently, weighted attention mechanisms are applied for feature fusion, focusing on the most relevant information. Finally, multi-layer perceptron is employed to accurately infer potential CMIs. In the fivefold cross-validation (CV) experiment on the baseline dataset, iHofman achieved an accuracy of 82.49% with an AUC of 0.9092. iHofman also demonstrates solid performance on other CMI datasets. In case studies, 26 of the top 30 CMIs with the highest iHofman predictive scores were confirmed in relevant literature.

**Conclusions:**

The above experimental results indicate that iHofman can effectively predict potential CMIs and has achieved outstanding performance compared with existing methods. It provides a reliable supplementary approach for subsequent biological wet experiments.

**Supplementary Information:**

The online version contains supplementary material available at 10.1186/s12915-025-02260-5.

## Background

With the increasing recognition of circular RNA (circRNA) as an endogenous non-coding RNA, it has attracted considerable interest from researchers in recent years. Unlike conventional linear RNAs, circRNA lacks a 5′ cap and 3′ poly-A tail, forming a single-stranded covalently closed structure [1]. Although circRNA was discovered in plant-like viruses over 40 years ago [2], it has often been regarded as a byproduct of abnormal RNA splicing [3] due to its less activity and the unknown nature of its biological function. Subsequently, circRNAs have been observed or synthesized in various animal virus [4], species, including prokaryotes [5], single-celled eukaryotes [6], and mammals [7]. Advances in high-throughput technology and specialized computational pipelines have restored the importance of circRNA in biological research. In particular, their covalently closed structure endows them with remarkable stability and a substantially longer half-life compared to linear RNAs [8], highlighting their potential significance in gene regulation and cellular physiology.

In 1993, Ambros and Ruvkun’s teams first identified miRNAs in *Caenorhabditis elegans* [9]. These small, single-stranded molecules are derived from the unique hairpin structure of transcripts, known as pre-miRNAs. Most miRNAs are initially transcribed from DNA sequences into primary miRNAs. These primary miRNAs subsequently undergo processing into precursor miRNAs, which are ultimately converted into mature miRNAs [10, 11]. Numerous experiments have shown that microRNA (miRNA) is also a single-stranded ncRNA, playing a crucial role in endogenous gene regulation and being an indispensable part of eukaryotic gene regulation [12, 13]. Recent studies indicate that circRNAs function as miRNA sponges [14–16] interacting with translation proteins driven by RNA-binding proteins. Notably, many circRNAs are dysregulated in cancer, where they contribute to tumor progression by disrupting normal miRNA and protein networks [17, 18].

CircRNAs have garnered extensive attention due to their unique closed-loop structure, stability, and diverse regulatory roles. A growing body of evidence indicates that circRNAs engage in complex associations with miRNAs, acting as sponges, decoys, or scaffolds to modulate miRNA activity and downstream gene expression [19]. These interactions are pivotal in fine-tuning cellular processes and can profoundly influence physiological and pathological states. For example, circ_0019693 promotes osteogenic differentiation of bone marrow mesenchymal stem cells (BMSCs) by regulating miR-942-5p, which targets Purkinje cell protein 4 (PCP4) and bone-coupled angiogenesis, thereby inhibiting the development of osteoporosis (OP) [20]. In ureteropelvic junction obstruction (UPJO) pathogenesis, circ_0045861 facilitates apoptosis and the progression of renal injury by interacting with miR-181 d-5p and modulating sirtuin 1 (SIRT1) expression [21]. Together, these studies underscore the nuanced and diverse roles that circRNAs play in regulating miRNA function across different tissues and disease models, which has profound implications for understanding gene regulation networks and disease mechanisms. While traditional biological studies have accurately explored the circRNA-miRNA relationship, these methods are time-consuming and labor-intensive [22]. Consequently, developing computational approaches to investigate circRNA-miRNA interactions (CMI) is of paramount importance. Such methodologies not only facilitate high-throughput analysis but also provide deeper insights into the functional implications of CMI in complex biological systems. Furthermore, CMI research is crucial in advancing cancer biology [23–25], as dysregulated circRNA and miRNA interactions are increasingly recognized as key contributors to tumorigenesis, metastasis, and treatment resistance. By applying computational models to large-scale expression and interaction datasets, researchers can pinpoint candidate biomarkers and therapeutic targets with greater speed and accuracy. Developing computational approaches to investigate circRNA-miRNA associations could significantly advance cancer biology research and provide valuable contributions to medical science. The integration of computational tools in CMI studies thus holds immense potential to drive innovation across medical sciences, offering new avenues for combating diseases and improving patient outcomes.

Recently, an growing number of computational models have been developed to predict associations among various biological and medical entities [26–28]. With advancements in computer technology, employing effective computational techniques to pre-screen a diverse range of potential candidates can greatly decrease the number of wet lab experiments needed, thereby accelerating research progress. For example, the study by Guo et al. [29] introduced CA-CMA, a model that combined circRNA and miRNA sequence features with CMIs. It constructed a molecular association network, optimized with labeled samples, and used a deep neural networks classifier for prediction. Ha et al. [30] introduced the DWMDA model, which proposed an application of deep graph neural networks to extract meaningful low-dimensional vectors using DeepWalk to identify miRNA-disease associations. Wang et al. [31] introduced JSNDCMI, the model combines the Denoising Autoencoder (DAE) with the multi-structure feature extraction framework to predict CMIs within sparse networks. JSNDCMI combines local topological structure similarity with functional similarity, using DAE for robust feature representation and a Gradient Boosting Decision Tree classifier was employed for prediction.

In addition, although most existing prediction methods have utilized representations of structure and sequence information [32–34], they only rely on static low-order representations and cannot dynamically highlight the most informative elements. For example, Liu et al. [35] introduced SSCRB, the model employs an attention mechanism to combine multi-scale sequence data with secondary structure features. Wei et al. [36] introduced DVMnet, which builds an enhanced De Bruijn graph and uses graph attention networks to capture latent structural features in RNA. Ha et al. [37] developed SMAP, which identifies miRNA-disease associations by applying the recommended algorithm and combining multiple similarity constraints of miRNA and diseases. While these approaches advance RNA prediction, they fail to separate and balance low-order and high-order sequence features, and cannot integrate comprehensive structural embeddings.

**Fig. 1:**
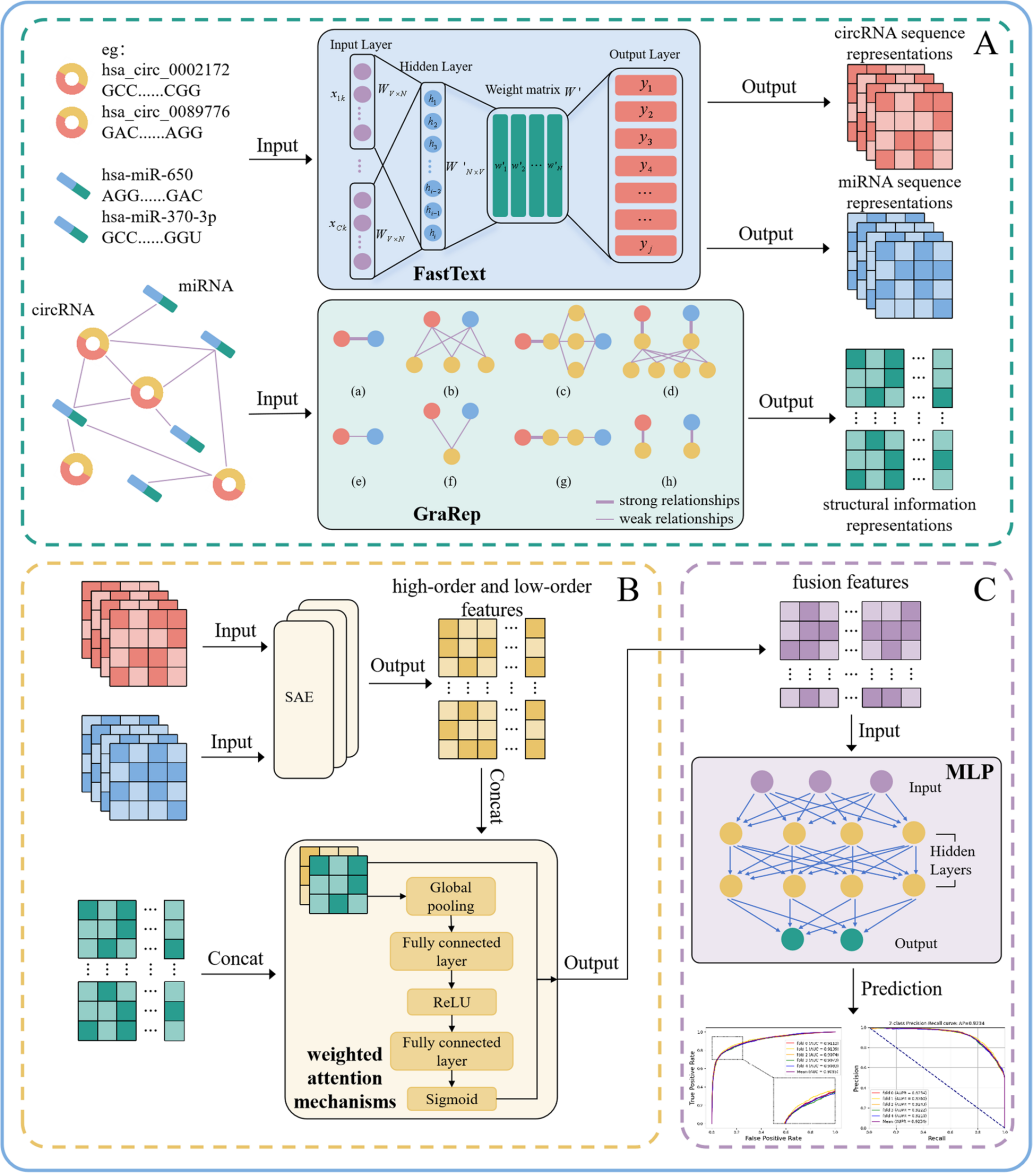
The flowchart of iHofman. **A** Extraction of low-order sequence information representations and structural information representations. **B** Extract high-order and low-order sequence information representations and integrate them with structural information representations. **C** Model training and prediction

To address the above issues, we introduce a novel approach called iHofman, which integrates multiple feature extraction techniques with weighted attention mechanisms to predict circRNA-miRNA interactions. The workflow of iHofman is illustrated in Fig. 1. Initially, iHofman constructs sequence information representations and structural information representations based on RNA sequences and CMI networks. Then, to effectively learn the high-order and low-order features of the sequence, SAE is employed to derive high-order and low-order features from the sequence information representations. Next, we fuse these features with the structural information representations using weighted attention mechanisms. Finally, the fusion feature matrix is input into the MLP classifier for training to predict potential CMIs. Experimental results show that iHofman can serve as an effective tool for accurately predicting potential CMIs. The source code and data can be freely downloaded from https://github.com/look0012/iHofman/.

The main contributions of this paper are as follows:

- iHofman adopts efficient feature extraction methods by combining FastText and GraRep to leverage both sequence and structural information. FastText excels at capturing subword-level features, which is crucial for handling the complexity and variability of circRNA and miRNA sequences. In parallel, GraRep applies graph-based representation learning to model structural relationships within the CMIs network, further improving the model’s ability to predict potential interactions.
- iHofman employs SAE to extract hierarchical feature representations, integrating both high-order and low-order features from sequential data. Low-level features identify basic patterns and local dependencies within the sequence. High-level features capture more abstract relationships. This dual-level feature extraction is essential for enhancing the prediction accuracy of CMIs. It enables the model to learn complex multidimensional data relationships.
- iHofman employs a weighted attention mechanism to combine multiple feature representations, including sequence-based features and structural data. Traditional models typically depend on static or equal-weight fusion strategies, which can overlook critical features or fail to adjust to varying feature subtleties. In contrast, our weighted attention mechanism dynamically shifts focus to the most relevant features, enhancing overall predictive performance. This adaptive fusion process enhances the model’s robustness and accuracy in identifying significant CMIs.

## Methods

### Dataset

To assess the predictive power of the iHofman model for CMI, we selected three widely used data sets that are representative of CMI, ensuring a fair and comprehensive assessment. The first dataset, CMI-9589 from the circbank database [38], includes 2115 circRNAs and 821 miRNAs, forming 9589 high-confidence association pairs. The second dataset, CMI-9905 [39], includes 9905 validated CMIs, combining high-confidence data from the circbank database with experimentally verified data from the circR2 Cancer database. It features interactions between 2346 circRNAs and 962 miRNAs. The third dataset, CMI-20208 [40], includes 20,208 high-confidence CMIs, involving a comprehensive set of interactions of 3569 circRNAs and 1152 miRNAs. A summary of the three datasets is provided in Table 1. In iHofman, these datasets are stored in matrix A in the form of an association matrix, where $A(i,j) = 1$ indicates that circRNA $c(i)$ is correlated with miRNA $m(j)$, and $A(i,j) = 1$ indicates no correlation.

**Table 1:** Details of the three datasets

Dataset	CMI-9589	CMI-9905	CMI-20208
CircRNA	2115	2346	3569
MiRNA	821	962	1152
Interactions	9589	9905	20,208

### Sequence information representation

This study utilized the FastText framework developed by Facebook AI Research [41] to encode circRNA and miRNA sequences into continuous vector representations. These embeddings simultaneously capture the overall sequence context and the composition of its subsequences. By integrating subword information into the Skip-Gram model of Word2 Vec, FastText is especially well suited for non-coding RNAs, since motifs of varying lengths convey critical functional signals. By decomposing each RNA sequence into overlapping *k*-mers and learning embeddings for these subwords, FastText ensures that even rare, biologically important motifs receive rich vector representations.

In the Skip-Gram model, each word is set as the central word and influenced by its neighboring words, necessitating multiple adjustments and predictions. Given base sequences $n_{1} ,n_{2} , \ldots$, maximizing the logarithmic likelihood is the objective of the Skip-Gram model.

1 $$\sum\limits_{t = 1}^{T} {\sum\limits_{ - c \le j \le c,j \ne 0} {\log } } p(n_{t + j} n_{j})$$

where the context refers to the set of indices surrounding the circRNA or miRNA sequence $n_{j}$. $T$ represents the number of bases sequences in circRNAs and miRNAs, and $c$ denotes the size of the context window. The probability $p(n_{t + j} |n_{j} )$ is defined as:

2 $$p(n_{t + j} n_{j} ) = \frac{\mathrm{exp} \left(\langle\nu^{\prime}_{nj+1},\nu_{nj} \rangle\right)}{\sum\limits_{n=1}^{N}\left(\langle\nu^{\prime}_{nj+1},\nu_{nj} \rangle\right)}$$

where $v_{n}$ and $v^{\prime}_{n}$ denote the input and output embeddings of the *n*th subword in ${\mathbb{R}}^{d}$. $N$ is the number of unique motifs in the training data, and $\left\langle { \cdot , \cdot } \right\rangle$ indicates the Euclidean inner product. In practice, when $N$ is large, performing a complete softmax calculation for all motifs is computationally impractical. To address this, we apply negative sampling, reformulating the objective to maximize a binary logistic loss:

3 $$\log \left( {\left\langle {v^{\prime}_{nj+i} ,v_{nj} } \right\rangle } \right) + \sum\limits_{i = 1}^{k} {E_{{n_{i} \sim P_{n} (w)}} } \left[ {\log \sigma \left( { - \left\langle {v^{\prime}_{nj+i} ,v_{nj} } \right\rangle } \right)} \right]$$

where $\sigma (x) = 1/(1 + e^{ - x} )$ is the sigmoid function, $n_{o}$ is an observed context, and the expectations run over $k$ negative samples drawn from a smoothed unigram distribution $P_{n} (w)$. This formulation drastically reduces training time while preserving the quality of learned embeddings.

4 $$sigma(x) = \frac{1}{1 + \exp ( - x)}.$$

The model incorporates all subword information in its input, and the output layer is a softmax classifier. The weight matrix $W^{\prime}$ acts as the context vector matrix. Probabilities are computed by taking the dot product of the central word vector and $W^{\prime}$. By employing gradient descent and cross-entropy loss, the word vectors are updated, resulting in the weight matrix $W$ connecting the input and hidden layers, which represents the trained central word embeddings.

### Structural information representations

In this study, we utilize the graph representation learning model GraRep to construct the structured information representation of CMI matrix. GraRep [42] is designed to learn vector representations of nodes, capturing global structural information in the network. In representation learning, GraRep distinguishes between neighbors across different node orders and can be extended to capture neighbors of any order.

GraRep builds on LINE by utilizing matrix factorization to capture *k*-order relational vector representations for network nodes. It captures k-order relational information of nodes and combines these k-order relationship vectors to produce the final result. The node degree matrix $D$ for network $G$ is derived from the adjacency matrix $S$.The following defines the probability transition matrix:

5 $$A^{k} = \underbrace {A \cdots \cdots A}_{k},A = D^{ - 1} S$$

where $(i,j)$ entry $A_{i,j}^{k}$ measures the probability that a random walker starting at $i$ reaches $j$ in exactly $k$ steps, the choice of maximum order $k$ is determined via cross-validation on held-out circRNA-miRNA interactions. Next, we treat each row of $A^{k}$ as the empirical distribution over context vertices $j$ for a target vertex $i$. Drawing inspiration from the Skip-Gram model, we interpret the observed *k*-step co-occurrences as positive samples and generate negative samples by drawing from the marginal distribution of vertices. Concretely, for each $(i,j)$ pair, we define the shifted positive log‐probability:

6 $$Y_{i,j}^{k} = \log \left( {\frac{{A_{i,j}^{k} }}{{\sum\nolimits_{h} {A_{j,h}^{k} } }}} \right) - \log \beta , \beta = \frac{\lambda }{|E|}$$

where $\lambda$ represents the number of negative samples for each positive example, and $|E|$ is the total number of edges in the CMIs graph. Intuitively, $Y_{i,j}^{k}$ measures the degree of surprise in observing $j$ at location $i$, relative to chance. Negative values of $Y_{i,j}^{k}$ imply the co-occurrence is no more likely than random; we therefore replace them with zero, yielding:

7 $$X_{i,j}^{k} = \max (Y_{i,j}^{k} ,0)$$

So that only statistically meaningful proximities contribute to the embedding. We then assemble for each order *k* the nonnegative matrix $X^{k} \in {\mathbb{R}}^{|V| \times |V|}$ and perform a rank-$d_{k}$ singular value decomposition

8 $$X^{k} U^{k} \Sigma^{k} (V^{k} )^{T}$$

where $U^k,V^k\;\in\mathbb{R}^{|v|\times d_k}$ are orthonormal and $\Sigma^{k} \in {\mathbb{R}}^{{d_{k} \times d_{k} }}$ contains the top $d_{k}$ singular values, and $\Sigma$ denotes the diagonal matrix. From this decomposition we extract two complementary embeddings:

9 $$H^{k} = U^{k} \left( {\Sigma^{k} } \right)^{\frac{1}{2}}$$

10 $$Z^{k} = V^{k} \left( {\Sigma^{k} } \right)^{\frac{1}{2}}$$

The representation of the current node $W_{k}$ is used as its column vector, while the representation of the context vertex $Z$ is also used as its column vector. Ultimately, GraRep provides feature vectors for all circRNA and miRNA in the data, capturing both local and global structural information.

### Feature extraction

To obtain high-order and low-order features of sequence information, we use stacked autoencoder (SAE) to extract these representations. SAE is a deep learning model constructed by stacking multiple autoencoders (AEs). SAE extracts hidden features from raw data layer by layer, progressively integrating them into a comprehensive representation. The working mechanism of an SAE lies in its hierarchical structure. This encoding process allows the model to learn compact and meaningful representations by reducing noise and preserving salient information. The decoder, in turn, reconstructs the original data from the hidden layer, ensuring that the learned features retain sufficient information about the input.

The key to extracting high-order and low-order features lies in the depth and iterative training of the SAE. Lower layers in the encoder primarily capture low-order features, which represent basic patterns or local dependencies in the sequence. As the data passes through deeper layers, the SAE captures high-order features, which represent more abstract, global relationships and long-term dependencies within the sequence. This hierarchical feature extraction enables the model to represent complex data structures effectively. When the feature vector $M$ of the raw data is input into an AE, the hidden layer is defined as follows:

11 $$Y^{l} = \sigma (M^{l} X^{l - 1} + b^{l} )$$

where $Z^{0} = X$, $M^{l} \in {\mathbb{R}}^{{h_{l} \times h_{l - 1} }}$ is the weight matrix, $b^{l} \in {\mathbb{R}}^{{h_{l} }}$ its bias vector, and $\sigma(\cdot)$ the element-wise sigmoid activation. We use a funnel architecture with layer sizes of 256, 128, and 64. The first layer condenses raw inputs into local motif features, the second extracts mid-level structural relationships, and the third captures overall regulatory patterns. Each weight matrix $M^{l}$ is initialized with Glorot uniform sampling and subsequently updated via the Adam optimizer. The learning rate is set to 10^−3^, and we apply early stopping based on a held-out validation set of 10% of the training samples, ensuring that the reduced representations retain the information essential for CMI analysis.

In each autoencoder layer, the decoder replicates the architecture of the encoder to reconstruct the original input from its latent representation.

12 $$\hat{Z}^{l - 1} = \sigma \left( {(M^{(l)} )^{T} Y^{l} + b^{^{\prime}l} } \right)$$

where $b^{{}^{\prime}\text{l}} \in \mathbb{R}^{h_{l-1}}$ is the decoder bias. The reconstruction loss at layer $l$ is defined as the mean squared error.

13 $${\mathcal{L}\ominus }^{l} = \frac{1}{n}\sum\limits_{i = 1}^{n} {\left\| {Z_{i}^{l - 1} - \hat{Z}_{i}^{l - 1} } \right\|_{2}^{2} }$$

By stacking three such autoencoders, we perform greedy layer-wise training: after training layer $l$ to minimize ${\mathcal{L}\ominus }^{l}$, we fix its weights and use $Y^{l}$ as input $Z^{l}$ to layer $l + 1$. This hierarchical process first captures short-range nucleotide patterns in circRNA, then identifies mid-range structural motifs linked to miRNA binding, and finally uncovers long-range dependencies indicative of global circRNA-miRNA regulatory modules. The final hidden layer output $Y^{3} \in {\mathbb{R}}^{n \times 64}$ encodes integrated representations that combine circRNA structural context with miRNA expression dynamics. In this experiment, SAE incorporates a dense layer composed of 256 and 128 units respectively, along with a bottleneck layer containing 64 units, and undergoes 20 epochs of training.

### Feature fusion 

In this study, we adopt a weighted attention mechanisms [43, 44] to fuse the circRNA sequence represents matrix, miRNA sequence represents matrix, and structural represents matrix to enhance model performance. We begin by defining a weighted attention layer that computes attention scores over the input features and re-weights them accordingly. Concretely, let the three input matrices be:

14 $$X_{circ} \in {\mathbb{R}}^{{n_{{{\text{circ}}}} \times d_{circ} }}$$

15 $$X_{mi} \in {\mathbb{R}}^{{n_{mi} \times d_{mi} }}$$

16 $$X_{interact} \in {\mathbb{R}}^{{n_{{{\text{circ}}}} \times n_{mi} \times d_{{_{interact} }} }}$$

where the circRNA sequence information representation is represented by $X_{circ}$, the miRNA sequence information representation is represented by $X_{mi}$, the CMI structural information representation is represented by $X_{interact}$, the number of circRNAs is represented by $n_{circ}$, the number of miRNAs is represented by $n_{mi}$, the feature dimension of circRNAs is denoted by $d_{circ}$, the feature dimension of miRNAs is denoted by $d_{mi}$, and the dimension of the structural information representations is denoted by $d_{interact}$. We use weighted attention mechanisms to fuse these features. For the circRNA and miRNA feature matrices, we calculate the attention weights, $a_{circ}$ and $a_{mi}$, respectively, through a fully connected layer.

17 $$a_{{\text{circ }}} = {\text{softmax}} \left( {W_{{a_{{\text{circ }}} }} X_{{\text{circ }}} + b_{{a_{{\text{circ }}} }} } \right)$$

18 $$a_{{\text{mi }}} = {\text{softmax}} \left( {W_{{a_{mi} }} X_{{\text{mi }}} + b_{{a_{{\text{mi }}} }} } \right)$$

where $W_{{a_{{\text{circ }}} }}$ and $W_{{a_{{{\text{mi}}}} }}$ are the learned weight matrices, and $b_{{a_{{\text{circ }}} }}$ and $b_{{a_{{\text{mi }}} }}$ are the bias terms, and the softmax operation ensures that $\sum\nolimits_{j} {a_{ * ,j} } = 1$ across the positions $j$. From a biological standpoint, this operation allows the network to highlight key nucleotide positions or structural segments in each molecule that significantly affect predictions, such as sequence motifs or structural protrusions related to binding or pairing.

Next, we apply these attention weights to re‐scale the original feature matrices element‐wise:

19 $$X_{{\text{circ,att }}} = X_{{\text{circ }}} \odot a_{{\text{circ }}}$$

20 $$X_{{\text{mi,att }}} = X_{{\text{mi }}} \odot a_{{\text{mi }}}$$

where $\odot$ denotes element‐wise multiplication broadcast across the feature dimension. This step effectively filters out noisy or less relevant features, sharpening the representation to focus on biologically salient signals.

Having obtained attended sequence embeddings for both circRNA and miRNA, we concatenate the weighted feature matrix with the structure embedding matrix.

21 $$C_{{\text{circ }}} = \left[ {X_{{\text{circ,att }}} ,X_{{\text{interact }}} } \right]$$

22 $$C_{{\text{mi }}} = \left[ {X_{{\text{mi,att }}} ,X_{{\text{interact }}} } \right]$$

Recognizing that different modalities may contribute unevenly to the final decision, we apply a second layer of attention to these concatenated features:

23 $$C_{{\text{circ,att }}} = C_{{\text{circ }}} \odot {\text{softmax}} \left( {W_{{a_{{\text{circ,final }}} }} C_{{\text{circ }}} + b_{{a_{{\text{circ,final }}} }} } \right)$$

24 $$C_{{\text{mi,att }}} = C_{{\text{mi }}} \odot {\text{softmax}} \left( {W_{{a_{{\text{mi,final }}} }} C_{{\text{mi }}} + b_{{a_{{\text{mi,final }}} }} } \right)$$

where $W_{{a_{{\text{circ,final }}} }}$ and $W_{{a_{{\text{mi,final }}} }}$ are the final attention weight matrices, $b_{{a_{{\text{circ,final }}} }}$ and $b_{{a_{{\text{mi,final }}} }}$ are the final bias terms.

Finally, we compute the fully fused representation by applying these attention scores and concatenating the modality outputs:

25 $$N_{output} \, = \left[ {C_{{\text{circ,att }}} ,C_{{\text{miRNA,att }}} } \right]$$

Using the weighted attention mechanisms, we effectively fuse the circRNA sequence information representations, miRNA sequence information representations, and structural information representations. This approach captures more complex biological information and enhances model performance. The experiment is conducted by using two 64-dimensional encoding vectors and connecting them with two behavioral vectors. Meanwhile, the second attention module is applied to combine the two outputs into a joint representation.

### Prediction and optimization

The feature vector processed by the weighted attention mechanism model serves as the input for the multi-layer perceptron (MLP), which then predicts CMI. The MLP is a powerful neural network composed of multiple layers of interconnected neurons, essential for extracting complex patterns from input data. The MLP structure was optimized through iterative experimentation and is defined as follows:

26 $$\hat{y}_{ij} = W^{l} \sigma^{l} \left( {W^{l - 1} \sigma^{l - 1} \left( { \cdots \left( {W^{2} \sigma \left( {W^{1} E + b^{1} } \right) + b^{2} } \right) \cdots } \right) + b^{l - 1} } \right) + b^{l}$$

where $W$ and $b$ refer to the weight matrix and bias vector in the *i*th layer, respectively. The *i*th layer’s activation function is denoted. $E$ is the concatenated embedding vector of circRNA *i* and miRNA at the *i*th layer. To avoid oversaturation, we chose to use the ReLU function. The final layer’s activation function is set to the sigmoid function, ensuring the model’s output is constrained between 0 and 1. Set the L2 regularization parameter to 0.0001 and run the iteration for 5000 times.

To train the model, the loss function of the model is defined as binary cross entropy, which can be expressed as follows:

27 $$Loss(y,\hat{y}) = - \frac{1}{N}\sum\limits_{i = 1}^{N} {[y_{i} \log (\hat{y}_{i} ) + (1 - y_{i} )\log (1 - \hat{y}_{i} )]}$$

where $y$ indicates the true labels, $\hat{y}$ denotes the model’s predicted labels, and $N$ represents the number of samples. By minimizing the loss using the Adam optimizer, the network is guided to assign high probabilities to known interaction pairs while reducing false positives. In essence, this loss function represents the Kullback–Leibler divergence between the empirical label distribution and the Bernoulli output of the model. Each update step helps align the model feature fusion mechanism with the potential biological relationship between circRNA and miRNA.

### Time and space complexity analysis

In this section, we analyze the time and space complexities of each module in the iHofman Model and provide the overall complexity.

Let $R_{c}$ and $R_{c}$ represent the counts of circRNA and miRNA records, each with *C* columns, while $M_{c}$ and $M_{m}$ represent the counts of their sequences. Creating embeddings for all sequences, using a shared vocabulary of size $E$ and dimension $D$, takes time proportional to $O\left( {(M_{c} + M_{m} )PD} \right)$, where $P$ is the padded length. For $N$ candidate circRNA-miRNA pairs, scanning each sequence’s embeddings with $E_{c}$ and $E_{m}$ results in a time complexity of $O(N \times (E_{c} + E_{m} ) \times D)$, and memory usage proportional to $N \times H$.

Next, graph-based features are extracted over *B* nodes with *H*-dimensional attributes. This adds time complexity proportional to $O((N/K)((E_{c} + E_{m} )D + BH))$ and memory usage proportional to $O(N \times H)$. During the k-fold CV, regenerating features takes $O((N/K)((E_{c} + E_{m} )D + BH))$ time. Two stacked autoencoders are then trained, with the training cycle number $E_{sae}$ and batch size $B_{size}$, contributing time complexity of $O(E_{sae} (N/K)F)$ and additional computation time of $O((N/K)F \cdot 256)$.

The attention fusion block costs $O((N/K)d)$, where $d \approx 2(64 + H)$, followed by standardization in $O((N/K)d)$. The final hidden layer-MLP fitting takes $O(I\cdot(N/K)dC_{out} )$, with inference and metric computation time of $O(I\cdot(N/K)dC_{out} )$. The total running time and peak memory size, after summing across all folds, are as follows:

28 $$O((R_{c} + R_{m} )C + (M_{c} + M_{m} )PED + N((E_{c} + E_{m} )D + BH) + E_{sae} NF256 + Nd^{2} + INdC_{out} ))$$

29 $$O((R_{c} + R_{m} )C + (M_{c} + M_{m} )PD + N(D + H + d) + F256 + dC_{out} ))$$

In our model, the parameters $P = 64$, $F = 64$, $E_{sae} = 20$, $B_{size} = 128$, $I = 5000$, and $C_{out} = 100$ have already been set, and $D$, $H$, $E_{c}$, $E_{m}$, $B$, and $C$ are determined at runtime from the data. In our work, the model algorithm was implemented and run using PyCharm Community Edition 2021.1 × 64, and the server is equipped with an Intel Core i5-12600 KF CPU, an RTX 4060 Ti GPU and 16 GB of memory.

## Results

### Evaluation criteria

In this study, we used the CMI-9905 dataset and applied fivefold cross-validation (CV) to evaluate the predictive performance of the iHofman model. Among them, the data was split into training, test, and validation sets in an 80%, 10%, and 10% ratio, respectively. The evaluation metrics include Accuracy ($Acc.$), Sensitivity ($Sen.$), Specificity ($Spe.$), Precision ($Pre.$), and Matthews Correlation Coefficient ($MCC$). The definitions of these metrics are as follows:

30 $$Acc. = \frac{TP + TN}{{TP + TN + FP + FN}}$$

31 $$Sen. = \frac{TP}{{TP + FN}}$$

32 $$Spe. = \frac{TN}{{TN + FP}}$$

33 $$Pre. = \frac{TP}{{TP + FP}}$$

34 $$MCC = \frac{TP \times TN - FP \times FN}{{\sqrt {(TP + FP)(TP + FN)(TN + FP)(TN + FN)} }}$$

where TP (true positive) and TN (true negative) represent the number of correctly predicted positive and negative samples by iHofman, respectively. Conversely, FP (false positive) and FN (false negative) indicate the number of incorrectly predicted positive and negative samples. Additionally, we plotted the ROC (receiver operating characteristic) curve, PR (precision-recall) curve, AUC (area under the ROC curve), and AUPR (area under PR) for iHofman to evaluate model performance.

### Prediction performance

**Fig. 2:**
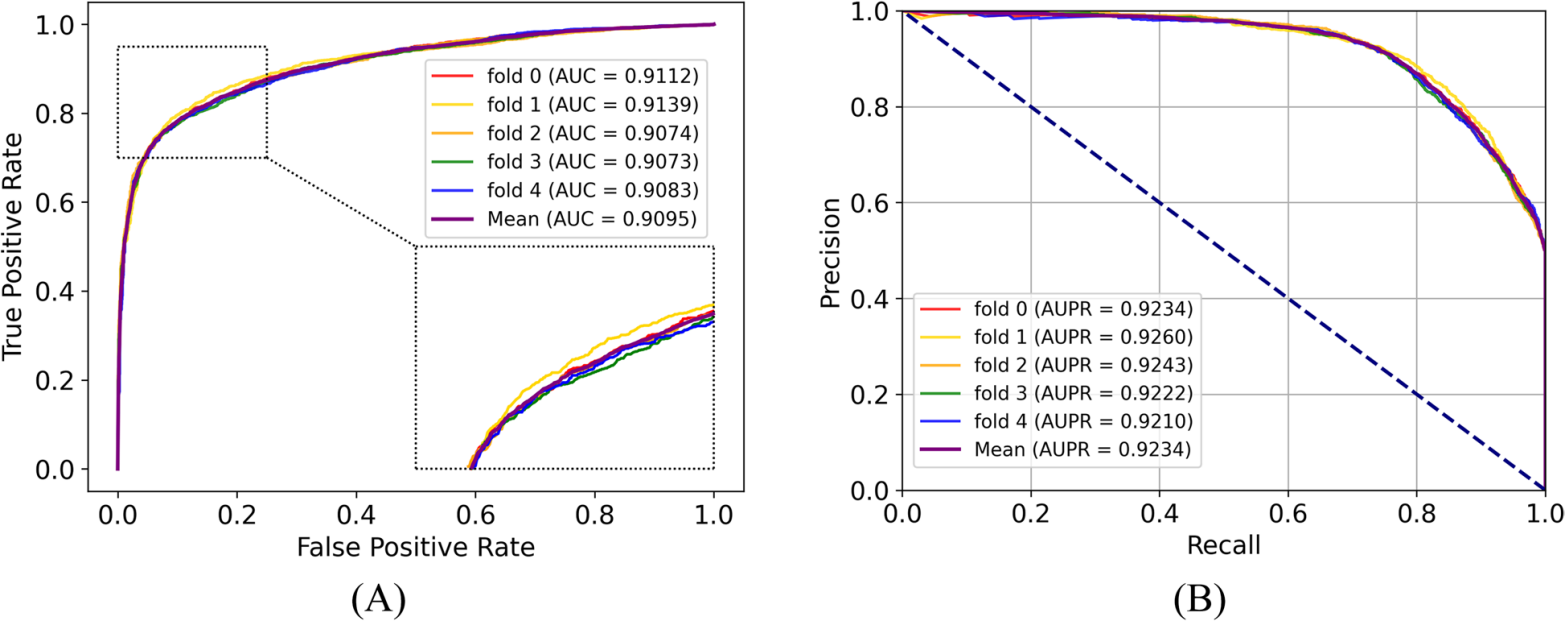
The ROC (**A**) and AUPR (**B**) of the fivefold CV based on CMI-9905 are shown. **A** The AUC was calculated by summing the areas under the ROC curve in panel **A**. **B** AUPR represents the area under the curve formed by precision and recall

In this evaluation, we use the CMI-9905 dataset to evaluate our model by fivefold CV. Table 2 presents the detailed evaluation metrics, with average results highlighted in bold. iHofman achieved an overall AUC of 0.9095, with individual fold AUCs of 0.9112, 0.9139, 0.9074, 0.9073, and 0.9083. The narrow spread of these metrics confirms the model’s high reproducibility. Specifically, overall accuracy varied by just 0.31%, while precision and the Matthews correlation coefficient fluctuated by 2.21% and 0.19%, respectively. Although sensitivity and specificity show slightly larger fluctuations of 2.85% and 2.27%, respectively, which may be reflected by the natural heterogeneity of positive and negative samples in each fold, these changes are still modest. Most strikingly, the standard deviations for AUC and AUPR are 0.0013 and 0.0009, respectively. Overall, these minimal deviations underscore the method’s strong performance and consistent behavior, highlighting its robustness and practical reliability. Figure 2 illustrates the ROC and precision-recall curves, from which we derive the AUC and AUPR values. Overall, the results indicate that iHofman delivers superior accuracy in predicting CMIs. Precise prediction of potential CMIs offers valuable insights into circRNA-miRNA interactions.

**Table 2:** The outcomes of the fivefold CV conducted on CMI-9905

5-Fold	Acc. (%)	Sen. (%)	Spe. (%)	Pre. (%)	MCC. (%)	AUC	AUPR
1	83.22	71.48	94.95	93.40	68.34	0.9112	0.9234
2	83.32	71.83	94.80	93.25	68.46	0.9139	0.926
3	83.72	74.46	92.98	91.39	68.63	0.9074	0.9243
4	83.75	77.03	90.46	88.98	68.11	0.9073	0.9222
5	83.95	77.64	90.26	88.85	68.44	0.9083	0.9210
**Average**	**83.59**	**74.49**	**92.69**	**91.17**	**68.40**	**0.9095**	**0.9234**
SD	0.31	2.85	2.27	2.21	0.19	0.0013	0.0009

### Comparison of different feature extraction strategies

**Fig. 3:**
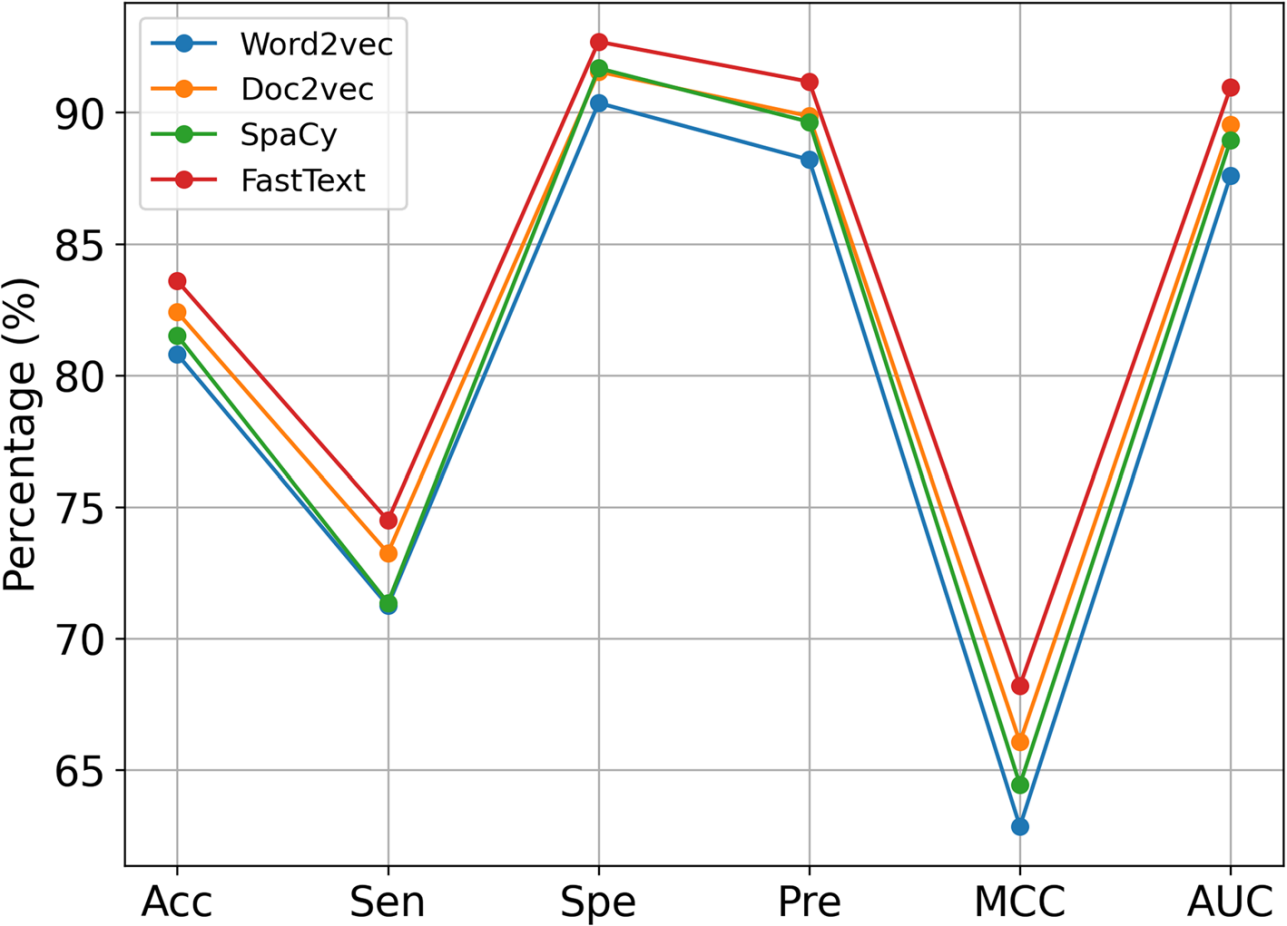
Results of fivefold CV acquired by different word embedding models

To evaluate the performance of the proposed model for extracting sequence information representations, we compared FastText with three word embedding methods: Word2vec, Doc2vec, and SpaCy. To ensure fairness and consistency in the experiment, we replaced the word embedding method in the proposed model with the sequence information representations generated by each of the three word embedding methods, while maintaining all other components of the model unchanged. We conducted the same fivefold CV experiment on the same dataset, with the comparison results presented in Table 3, with the averages obtained by our model highlighted in bold. To facilitate a more intuitive comparison, a line chart is presented in Fig. 3. As illustrated in Table 3 and Fig. 3, the FastText method outperforms the other three word embedding methods. Specifically, the iHofman model attained higher accuracy, sensitivity, specificity, precision, MCC, and AUC by 1.19%, 1.24%, 1.14%, 1.31%, 2.34%, and 0.0142, respectively, compared to the next highest, Doc2vec. In summary, the word embedding method in our model, FastText, is better suited for extracting sequential information representations, possibly because the ability of FastText to generate subword-level representations enables it to capture more fine-grained information. While methods such as Word2vec are powerful, they may not take full advantage of morphological differences in words, which can limit their performance in tasks involving complex structures.

**Table 3:** Results of the fivefold CV obtained with different word embedding methods

Feature extraction	Acc. (%)	Sen. (%)	Spe. (%)	Pre. (%)	MCC (%)	AUC
Word2vec	80.80 ± 0.76	71.25 ± 3.72	90.36 ± 2.37	88.20 ± 2.03	62.85 ± 0.89	0.8760 ± 0.0015
Doc2vec	82.40 ± 0.45	73.25 ± 3.96	91.55 ± 3.10	89.86 ± 2.91	66.06 ± 0.27	0.8953 ± 0.0027
SpaCy	81.51 ± 0.67	71.35 ± 3.17	91.68 ± 1.84	89.64 ± 1.65	64.44 ± 0.72	0.8895 ± 0.0014
FastText	**83.59 ± 0.31**	**74.49 ± 2.85**	**92.69 ± 2.27**	**91.17 ± 2.21**	**68.40 ± 0.19**	**0.9095 ± 0.0013**

### Comparison of fusion with different features

**Fig. 4:**
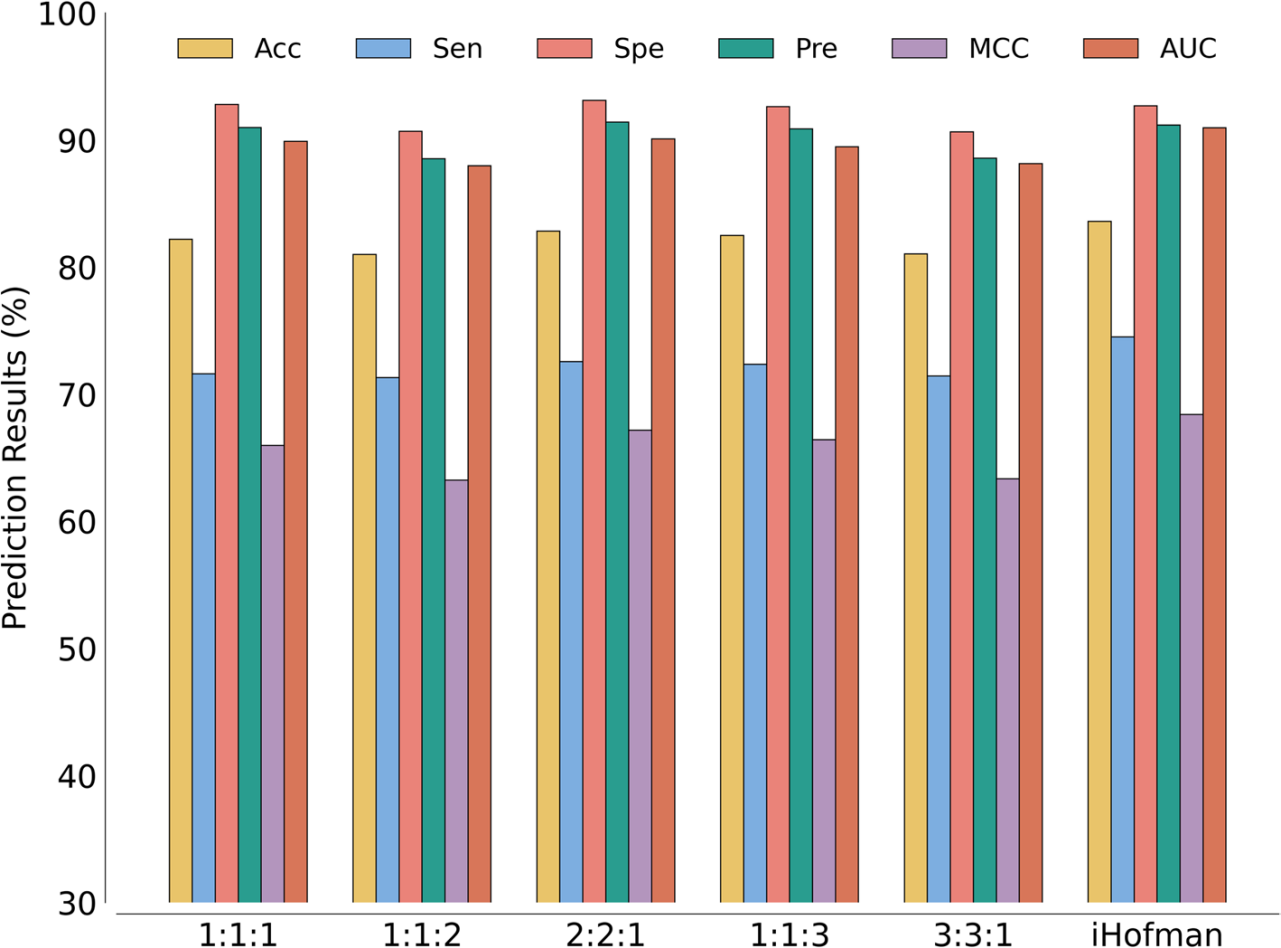
Bar graph showing various feature fusion proportions

iHofman employs weighted attention mechanisms for feature fusion, enhancing the model’s ability to extract and integrate valuable information from various data sources. This dynamic focus on different information segments improves overall task performance and efficiency. To evaluate the effectiveness of the weighted attention mechanism, we tested the feature fusion of three representations (circRNA sequence information representations, miRNA sequence information representations, and structural information representations) at different ratios: 1:1:1, 1:1:2, 2:2:1, 1:1:3, and 3:3:1. Because circRNA and miRNA sequence representations have equal importance, they were kept at the same proportion. All experiments used identical parameter settings to ensure a fair comparison. Table 4 presents detailed results and Fig. 4 shows bar graphs for each fusion ratio, indicating that the 2:2:1 combination delivered the best performance among static ratios. However, compared to weighted attention fusion, the static ratios improved only specificity by 0.41%, while all other metrics remained below the weighted attention results.

**Table 4:** Results of the fivefold CV obtained with varying feature fusion ratios

Feature fusion	Acc. (%)	Sen. (%)	Spe. (%)	Pre. (%)	MCC (%)	AUC
1:1:1	82.19 ± 0.75	71.60 ± 3.70	92.78 ± 2.22	90.97 ± 2.17	65.97 ± 0.69	0.8988 ± 0.0004
1:1:2	80.99 ± 0.68	71.30 ± 3.03	90.68 ± 1.94	88.52 ± 1.71	63.24 ± 0.93	0.8796 ± 0.0007
2:2:1	82.83 ± 0.75	72.56 ± 2.94	**93.10 ± 1.68**	90.39 ± 1.65	67.15 ± 1.01	0.9008 ± 0.0012
1:1:3	82.48 ± 0.25	72.34 ± 2.91	92.62 ± 2.45	90.87 ± 2.44	66.42 ± 0.32	0.8946 ± 0.0011
3:3:1	81.02 ± 0.68	71.42 ± 3.83	90.63 ± 2.73	88.56 ± 2.46	63.34 ± 0.84	0.8814 ± 0.0017
iHofman	**83.59 ± 0.31**	**74.49 ± 2.85**	92.69 ± 2.27	**91.17 ± 2.21**	**68.40 ± 0.19**	**0.9095 ± 0.0013**

**Fig. 5:**
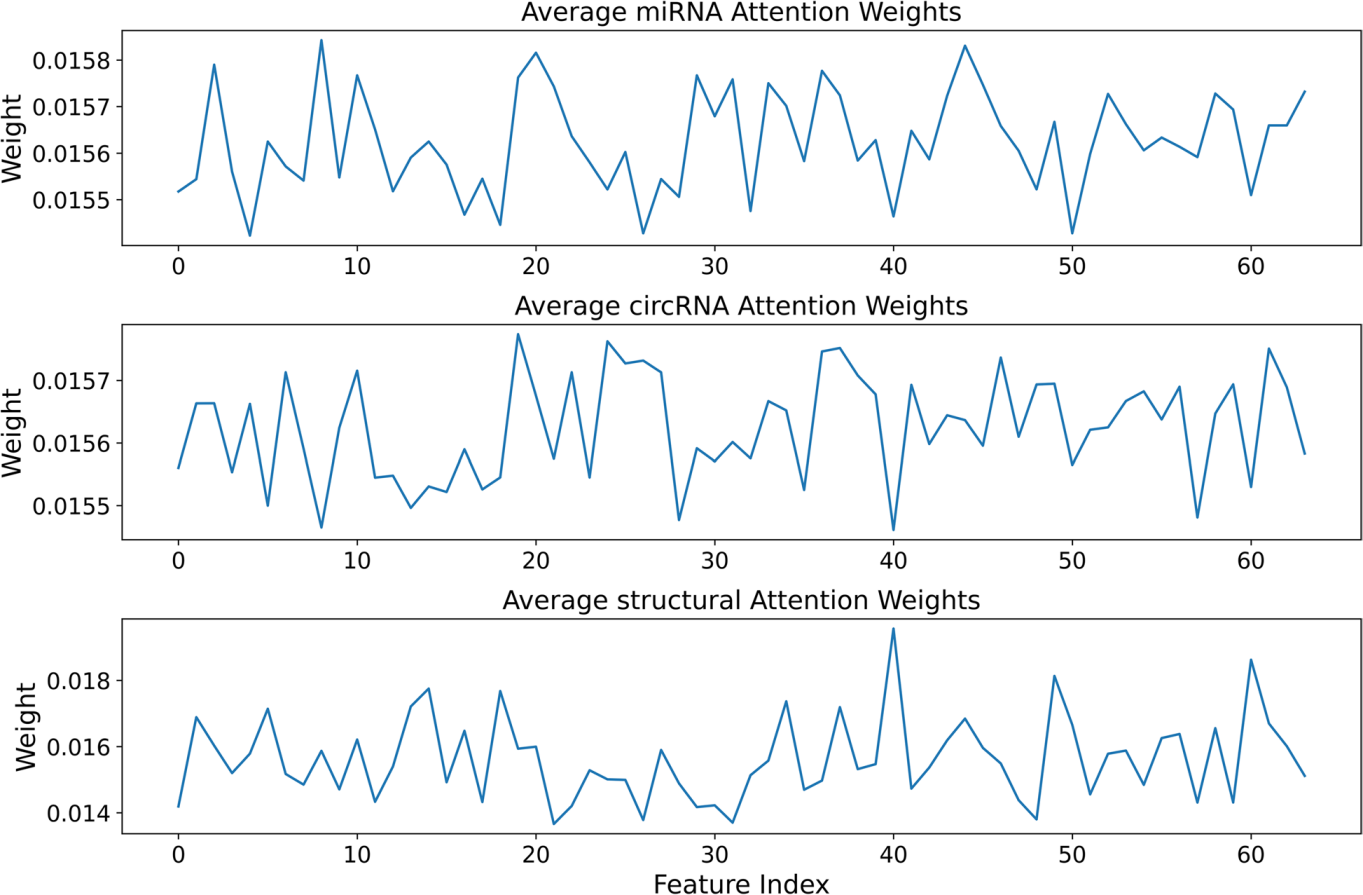
Weighted attention mechanism integrates the weight distributions of different features

Furthermore, we conducted an in-depth analysis of the weighted attention distribution among the features. Figure 5 shows the detailed distribution of the attention weights for the three types of features. This figure shows the different weight proportions of each feature in the 64-dimensional space, and the sum of all proportions is 1. We also computed the average attention weights for each feature, among which the average attention weight of the circRNA sequence information representations is 0.41, that of the miRNA sequence information representations is 0.38, and that of the structural information representations is 0.21. Although this distribution closely resembles the previously defined optimal static ratio of 2:2:1, Fig. 5 shows that the attention weights for each feature type are not evenly distributed. The weighted attention mechanism outperforms the static ratio-based fusion strategy by dynamically adjusting its focus on different features. The dynamic nature of the weighted attention allows the model to focus on the most relevant features without becoming overly dependent on fixed ratios. In conclusion, the weighted attention mechanism for feature fusion outperforms static feature combination by delivering better overall model performance through its dynamic adaptation to the complex features in the data.

### Comparison with other deep learning models

**Fig. 6:**
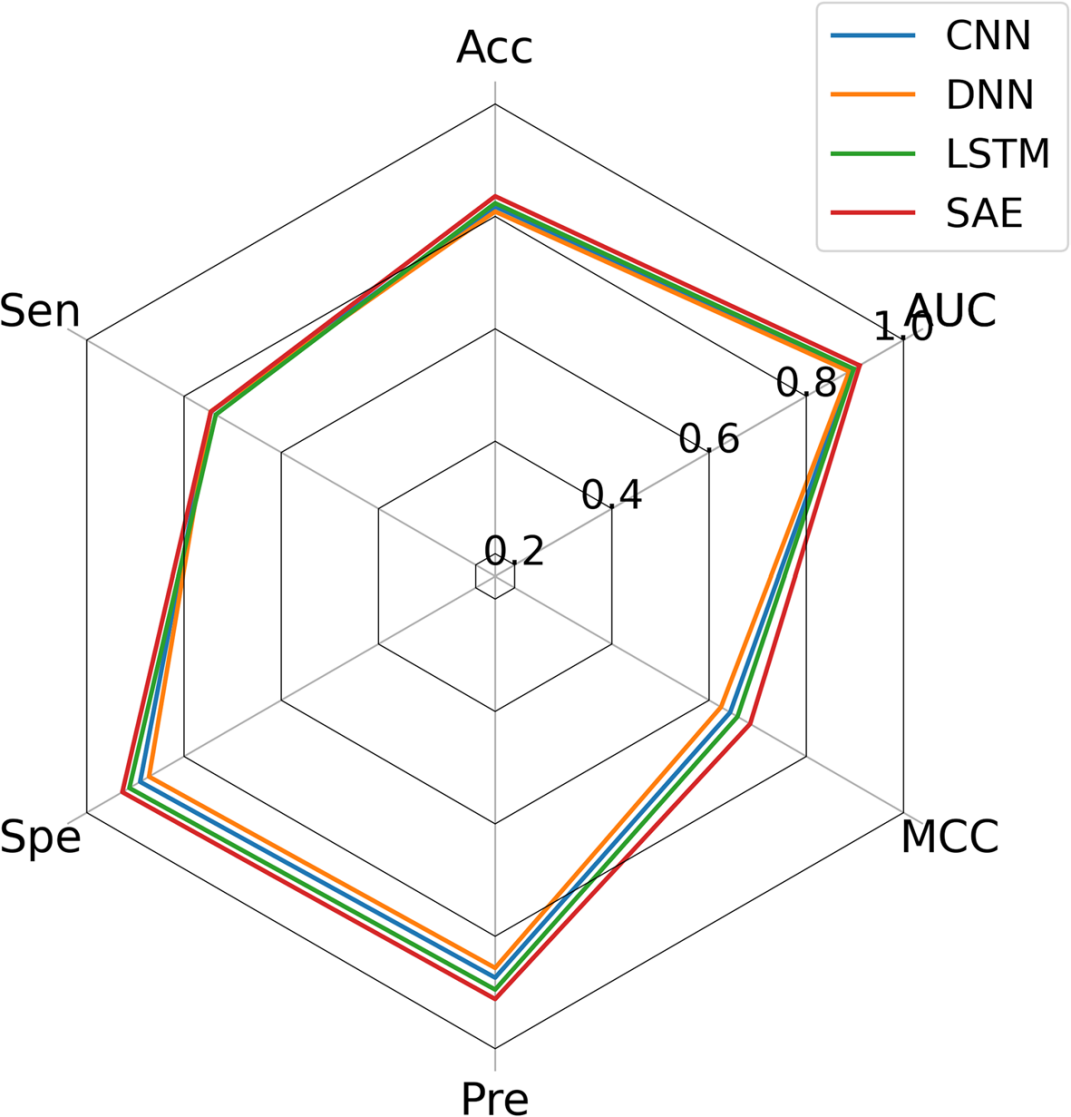
Radar plots of different models on various performances

Feature extraction algorithms significantly impact model performance. To ensure an equitable comparison, we evaluated different deep learning methods to determine if the SAE used in the proposed model enhances CMIs prediction performance. We selected three different models for comparison: convolutional neural network (CNN), deep neural network (DNN), and long short-term memory network (LSTM). The CNN model excels at capturing spatial patterns through convolutional filters applied across input matrices; the DNN serves as a versatile, fully connected baseline; and the LSTM is tailored to sequential data, capturing temporal dependencies via gated memory units. For fairness, we replaced the above models in the comparison, keeping all other parameters constant. The comparison results are shown in Table 5, with bold values indicating the mean results achieved by the SAE in our model during fivefold CV. For clearer comparison, a radar map of the results from Fig. 6 is provided. As shown in Table 5 and Fig. 6, the sensitivity scores of CNN, DNN, and LSTM are higher than those of the SAE model, with DNN achieving the highest sensitivity score, 0.04% higher than SAE. However, the SAE used in our model achieved the best results in accuracy, specificity, precision, MCC and AUC. Especially in terms of MCC and AUC, which are critical indicators of the overall performance and discriminative power of the model. Furthermore, to validate the effectiveness of SAE, we conducted a statistical test comparing SAE with the three aforementioned methods based on the AUC score. The resulting *p*-value of 7.359 × 10^−7^ (*p* < 0.05) confirms that the SAE-based model’s performance advantage over CNN, DNN, and LSTM is statistically significant. These findings indicate that SAE is the most effective feature extractor for CMI prediction among the models evaluated. This may be attributed to the advanced feature extraction capabilities of SAE, as it learns to compress feature spaces through dimensional reduction, which is particularly important for high-dimensional data. Dimensional reduction helps eliminate redundant features, reduces the impact of noise on model performance, and enables SAE to capture complex data features, providing high-quality input for subsequent prediction tasks.

**Table 5:** Results of the fivefold CV obtained with varying deep learning models

Model	Acc. (%)	Sen. (%)	Spe. (%)	Pre. (%)	MCC (%)	AUC
CNN	81.75 ± 0.79	74.47 ± 4.34	89.03 ± 3.53	87.36 ± 2.94	64.34 ± 1.26	0.8946 ± 0.0018
DNN	80.86 ± 0.30	74.53 ± 4.66	87.20 ± 4.40	85.63 ± 3.56	62.45 ± 0.71	0.8882 ± 0.0015
LSTM	82.35 ± 0.71	73.46 ± 3.51	91.24 ± 2.74	89.49 ± 2.52	65.85 ± 1.16	0.8975 ± 0.0022
SAE	**83.59 ± 0.31**	**74.49 ± 2.85**	**92.69 ± 2.27**	**91.17 ± 2.21**	**68.40 ± 0.19**	**0.9095 ± 0.0013**

### Comparison with different classifier models

In this section, we evaluate how different classifiers influence the performance of the iHofman framework. We used MLP classifiers as the baseline classification strategy for iHofman models. We maintained the same feature extraction and fusion methods, replacing only the MLP classifier with random forest (RF) [45], logistic regression (LR), support vector machine (SVM), *K*-nearest neighbor (KNN) [46], AdaBoost (AdaB), and gradient boosting (GB) algorithms.

**Fig. 7:**
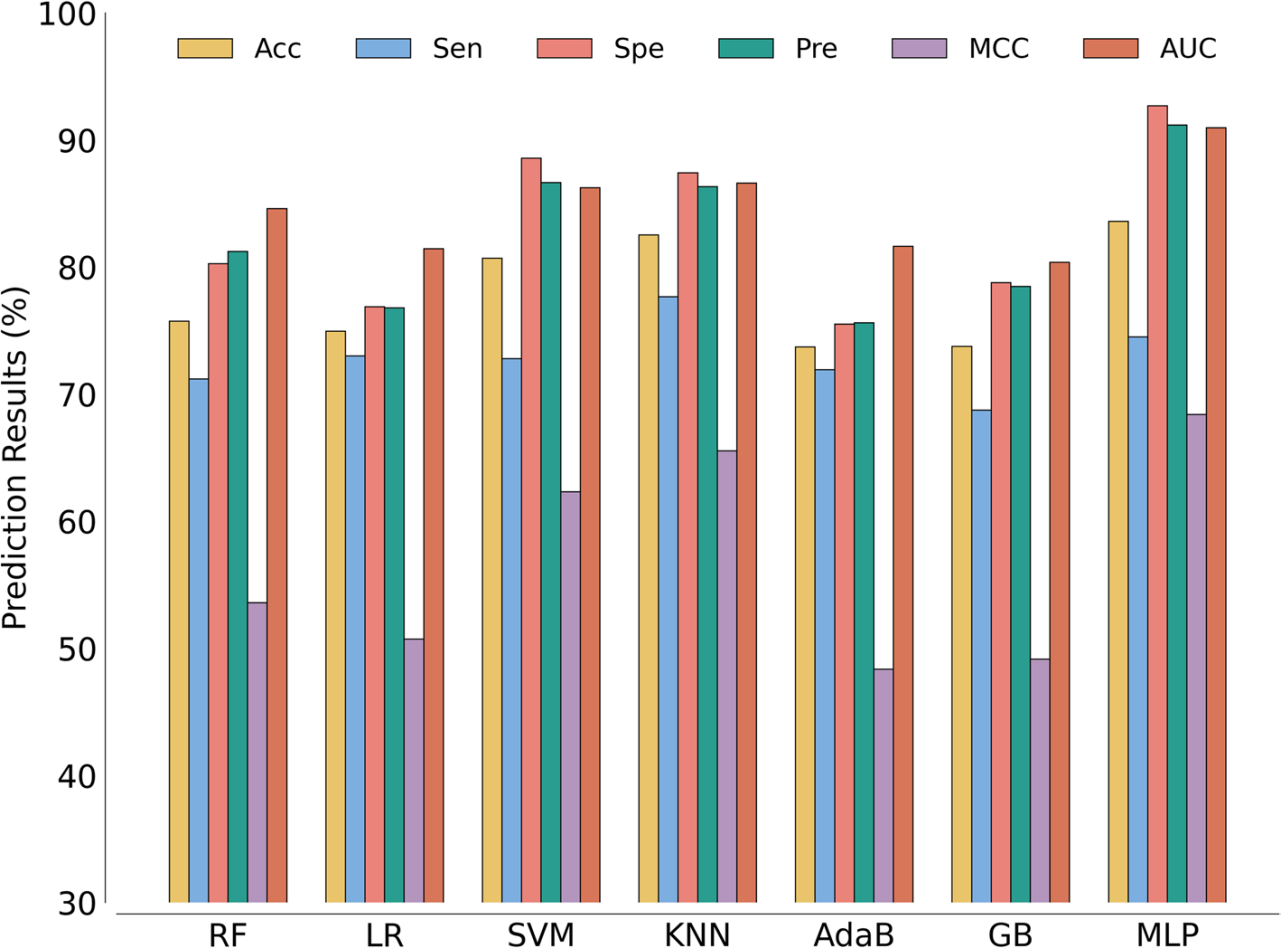
Bar graph comparing different classifier models

Table 6 presents the average results of these models on the same dataset using fivefold CV, with bold values indicating the average results achieved by our model. Figure 7 complements this with a histogram visualization, allowing for an immediate, side-by-side comparison of performance. The best average AUC values achieved were 0.9095 for MLP, 0.8459 for RF, 0.8143 for LR, 0.8624 for SVM, 0.8659 for KNN, 0.8163 for AdaB, and 0.8037 for GB. From Fig. 7, KNN achieves the highest sensitivity, but its accuracy, specificity, precision, MCC, and AUC are inferior to those of MLP classifiers. The lower accuracy, specificity, and precision observed with KNN suggest it may capture positive cases at the expense of overall prediction quality. In contrast, the MLP classifier demonstrates a balanced performance across all metrics, indicating it effectively minimizes both false positives and false negatives while maintaining robust overall classification accuracy. In summary, the MLP classifiers selected for the iHofman model demonstrate the best overall performance, outperforming other tested classifiers. We therefore conclude that the MLP remains the most suitable classification strategy for iHofman. Its capacity to learn complex, non-linear relationships within the fused feature space underpins its exceptional predictive reliability, making it the optimal choice for ensuring high performance in our CMI prediction framework.

**Table 6:** Results of the fivefold CV obtained with different classifier models

Classifier	Acc. (%)	Sen. (%)	Spe. (%)	Pre. (%)	MCC (%)	AUC
RF	75.73 ± 3.04	71.18 ± 13.58	80.26 ± 15.92	81.22 ± 11.60	53.58 ± 6.32	0.8459 ± 0.0009
LR	74.94 ± 1.26	73.01 ± 10.59	76.86 ± 9.64	76.78 ± 5.10	50.73 ± 1.99	0.8143 ± 0.0013
SVM	80.68 ± 0.67	72.80 ± 4.59	88.56 ± 3.63	86.65 ± 2.99	62.31 ± 0.89	0.8624 ± 0.0009
KNN	82.53 ± 1.03	77.66 ± 2.91	87.40 ± 4.88	86.33 ± 4.16	65.54 ± 2.51	0.8659 ± 0.0024
AdaB	73.71 ± 0.71	71.92 ± 10.69	75.51 ± 10.66	75.60 ± 5.69	48.35 ± 1.03	0.8163 ± 0.0020
GB	73.75 ± 1.64	68.74 ± 12.05	78.77 ± 13.77	78.47 ± 9.09	49.14 ± 3.77	0.8037 ± 0.0045
MLP	**83.59 ± 0.31**	**74.49 ± 2.85**	**92.69 ± 2.27**	**91.17 ± 2.21**	**68.40 ± 0.19**	**0.9095 ± 0.0013**

### Ability analysis of the model to deal with imbalanced data

In this section, we assessed model performance on the CMI-9905 dataset by varying the positive-to-negative sample ratio to evaluate its handling of class imbalance. We extended our experiments to five plus-negative ratios 1:1, 1:2, 1:3, 1:4, and 1:5, evaluating not only AUC and AUPR, but also accuracy, sensitivity, specificity, precision, *F*1 score, and Matthews correlation coefficient. The detailed results are shown in Table 7, under the balance condition of positive and negative ratio 1:1, the average AUC of iHofman is 0.9095, AUPR is 0.9234, *F*1 is 80.69%, MCC is 68.40%. With ratios of 1 to 2 and 1 to 3 the AUC remained above 0.9000, AUPR stayed above 0.8400 and MCC held at 72.20%. Even in the case of extreme imbalance of the dataset with a positive and negative ratio of 1:5, iHofman still maintains AUC of 0.9015, AUPR of 0.8006, *F*1 is 75.90%, and MCC is 73.36%. In the setting of only varying the proportion of positive and negative samples, iHofman consistently provides superior accuracy and AUC, and also clearly shows a robust balance between sensitivity and specificity on skewed data. These comprehensive results confirm that iHofman achieves stable, high-confidence predictions when dealing with imbalanced datasets.

**Table 7:** iHofman performance score on imbalanced datasets

Ratio	Acc. (%)	Sen. (%)	Spe. (%)	Pre. (%)	*F*1 (%)	MCC (%)	AUC	AUPR
1:1	83.59	74.49	92.69	91.17	80.69	68.40	0.9095	0.9234
1:2	87.73	70.42	96.39	90.73	79.29	71.92	0.9044	0.8719
1:3	90.12	67.12	97.79	91.02	77.25	72.48	0.9019	0.8411
1:4	91.74	65.23	98.36	90.90	75.95	72.55	0.9011	0.8177
1:5	93.10	65.20	98.68	90.83	75.90	73.36	0.9015	0.8006

### Comparison with other state-of-the-art models

With advancing research in CMI forecasting, numerous new forecasting methods have been proposed by distinguished researchers. To comprehensively demonstrate the performance of the iHofman model, we compared it across three datasets and eight state-of-the-art models using uniform AUC scoring parameters to ensure fairness. The datasets include CMI-9905, CMI-9589, and CMI-20208, and the models are KGDCMI [39], WSCD [40], SGCNCMI [47], JSNDCMI [31], DeepCMI [48], SJLD-CMI [49], KS-CMI [50], and CA-CMA [29].

We calculated the AUC and AUPR scores for each model using the CMI-9905 dataset, with summary results presented in Table 8. According to Table 8, the AUC and AUPR scores of iHofman are the highest, exceeding those of the second-best model KS-CMI by 0.0009 and 0.009, respectively. To further validate the model’s performance, a statistical test based on the AUPR score was conducted, resulting in a *P*-value of 0.0271 (*p* < 0.05). This indicates that the iHofman model is significantly better than the KS-CMI model. In addition, we also measured the running time of the model. MLP training proved highly efficient, requiring 5.49, 5.86, 6.52, 6.70, and 5.63 s for each fold, and averaging 6.04 s per fold. Data preprocessing, feature engineering, and evaluation overhead is 14.87 s per fold. In total, including feature processing and model fitting across all five folds, the process completed in 104.55 s.

**Table 8:** The AUC and AUPR scores obtained by the various models on the CMI-9905 dataset

Methods	WSCD	SGCNCMI	JSNDCMI	DeepCMI	BJLD-CMI	KS-CMI	iHofman
AUC	0.8923	0.8942	0.9003	0.9054	0.9069	0.9086	**0.9095**
AUPR	0.8935	0.8887	0.8999	0.8978	0.8920	0.9144	**0.9234**

To conduct a comprehensive evaluation of the iHofman model, we performed fivefold CV on the CMI-9589 and CMI-20208 datasets and statistically analyzed the detailed performance indicators, which are presented in Additional file 1: Table S1 and Table S2 respectively. The results for the CMI-9589 dataset are summarized in Table 9. Table 9 shows that on CMI-9589, the iHofman model’s AUC and AUPR scores exceed those of the second-best KS-CMI model by 0.0008 and 0.0088, respectively. In addition, model training averaged 5.95 s per fold. The average time per fold data preprocessing, feature engineering, and evaluation overhead is 14.21 s. Overall, including feature processing and model fitting across all five folds, the total runtime was 100.80 s.

**Table 9:** The AUC and AUPR scores obtained by the various models on the CMI-9589 dataset

Methods	SGCNCMI	KGDCMI	CA-CMA	KS-CMI	iHofman
AUC	0.9015	0.9041	0.9156	0.9179	**0.9187**
AUPR	0.9011	0.8937	0.9086	0.9181	**0.9269**

For the CMI-20208 dataset (only the WSCD and CA-CMA models are tested on the CMI-20208 dataset, so only the above two models are compared), the results for the CMI-20208 dataset are summarized in Table 10. The AUC and AUPR scores of the iHofman model are 0.0003 and 0.0080 higher than those of the suboptimal CA-CMA model, respectively. In addition, model training averaged 12.61 s per fold. Data preprocessing, feature engineering, and evaluation incurred an average overhead of 32.36 s per fold. Overall, the cumulative running time of all the 5 folds, including feature processing and model fitting, was 224.85 s. Overall comparisons across three datasets demonstrate that the iHofman model significantly outperforms existing methods in prediction accuracy.

**Table 10:** The AUC and AUPR scores obtained by the various models on the CMI-20208 dataset

Methods	WSCD	CA-CMA	iHofman
AUC	0.8898	0.9170	**0.9173**
AUPR	0.8847	0.9131	**0.9211**

To confirm model stability, we conducted paired Student’s *t*-tests on the fivefold cross-validated AUC and AUPR scores for each dataset; the detailed results appear in Table 10. As shown in Table 11, on the CMI-9905 dataset our approach achieved a mean AUC of 0.9095 with a standard deviation of 0.0013 and a mean AUPR of 0.9234 with a standard deviation of 0.0009. The paired *t*-test yielded *t* = 561.31 and two-tailed *p* = 6.04 × 10^−11^ for AUC and *t* = 634.67 and *p* = 3.70 × 10^−11^ for AUPR. Equally compelling results emerged on the CMI-9589 dataset where our method achieved a mean AUC of 0.9187 with a standard deviation of 0.0029 and the paired *t*-test yielded a *t*-statistic of 322.82 together with a two-tailed *p*-value of 5.52 × 10^−10^. On the same dataset, the mean AUPR reached 0.9269 with a standard deviation of 0.0031, *t* = 311.16 and *p* = 6.40 × 10^−10^. In the larger CMI-20208 dataset, our model achieved a mean AUC of 0.9174 ± 0.0026 with *t* = 357.02 and *p* = 3.69 × 10^−10^ and a mean AUPR of 0.9211 ± 0.0020 with *t* = 479.79 and* p* = 1.13 × 10^−10^. In three datasets, both one-tailed and two-tailed *p*-values fall well below 10^−9^, unequivocally demonstrating that the superior performance of our method is highly unlikely to arise by chance.

**Table 11:** Statistical analysis of AUC and AUPR for different datasets

Dataset	Metric	Mean	SD	*T*-statistic	*P*-value (two-sided)	*P*-value (one-sided)
CMI-9905	AUC	0.9095	0.0013	561.3111	6.04 × 10^−11^	3.02 × 10^−11^
	AUPR	0.9234	0.0009	634.6655	3.70 × 10^−11^	1.85 × 10^−11^
CMI-9589	AUC	0.9187	0.0029	322.8152	5.52 × 10^−10^	2.76 × 10^−10^
	AUPR	0.9269	0.0031	311.1649	6.40 × 10^−10^	3.20 × 10^−10^
CMI-20208	AUC	0.9173	0.0026	357.0180	3.69 × 10^−10^	1.85 × 10^−10^
	AUPR	0.9211	0.0020	479.7862	1.13 × 10^−10^	5.66 × 10^−11^

### Generalization analysis

To assess the generalization of the proposed iHofman model, we retrieved 4966 experimentally validated lncRNA-miRNA interaction pairs from the lncRNASNP2 database, encompassing 770 lncRNAs and 275 miRNAs with known sequences [51, 52]. We trained the iHofman model on this dataset and compared it with the benchmark algorithms in the field of lncRNA-miRNA interaction prediction. The detailed fivefold CV results are presented in Additional file 1: Table S3. As shown in Table 12, iHofman achieved the highest AUC and AUPR values of 0.9149 and 0.9214, respectively. It was 0.0498 higher in AUC and 0.0521 higher in AUPR than the second-best GATv2. This performance underscores the superior capability of iHofman to distinguish true interactions and its stability across different validation sets. Furthermore, in terms of accuracy, recall and *F*1 score, iHofman outperforms the GATv2 model. Only the precision is slightly lower than that of GATv2, while the recall has increased by 10.99% and the *F*1 score has risen by 3.87%. The above experimental results indicate that iHofman not only can accurately predict the interactions between circRNAs and miRNAs, but also achieved good results in the lncRNA-miRNA interaction dataset, thereby verifying the wide applicability and strong generalization performance of the iHofman model.

**Table 12:** The performance comparison between iHofman and baselines on lncRNA-miRNA interaction dataset

Method	AUC	AUPR	Acc. (%)	Pre. (%)	Rec. (%)	*F*1 (%)
NDALMA	0.8389	0.8516	79.81	77.81	77.13	71.34
preMLI	0.8104	0.8028	73.48	71.46	78.66	74.78
RNAI-FRID	0.8292	0.8468	75.29	76.16	73.68	74.88
GATv2	0.8651	0.8693	80.54	**82.07**	78.21	80.06
iHofman	**0.9149**	**0.9214**	**82.90**	79.38	**89.20**	**83.93**

## Case study

To validate the effectiveness of iHofman in identifying new CMIs, the case study was conducted using the CMI-9905 dataset. In this case study, the model was trained on known CMI data and subsequently applied to predict unknown CMIs. The predicted CMIs were then ranked by their scores and verified through relevant research literature or experiments. Among the top 30 predicted CMI pairs, only 4 were not verified by recent literature, as illustrated in Table 13. In conclusion, the case study results indicate that iHofman has exceptional predictive power for identifying potential CMIs, suggesting it could be applied to provide a robust preselection tool for biological experiments.

**Table 13:** Top 30 CMIs with the highest score predicted by iHofman

Rank	CircRNA	MiRNA	Evidence	Rank	CircRNA	MiRNA	Evidence
1	hsa_circ_0000554	hsa-miR-339-5p	Confirmed	16	hsa_circ_0010559	hsa-miR-2861	Unconfirmed
2	hsa_circ_0000592	hsa-miR-139-3p	Confirmed	17	hsa_circ_0012129	hsa-miR-661	Confirmed
3	hsa_circ_0001658	hsa-miR-181c-5p	Confirmed	18	hsa_circ_0023019	hsa-miR-4267	Confirmed
4	hsa_circ_0000799	hsa-miR-31-5p	Confirmed	19	hsa_circ_0026096	hsa-miR-4763-3p	Unconfirmed
5	hsa_circ_0000993	hsa-miR-214-5p	Confirmed	20	hsa_circ_0040863	hsa-miR-6756-5p	Confirmed
6	hsa_circ_0001427	hsa-miR-181c-5p	Confirmed	21	hsa_circ_0041103	hsa-miR-103a-3p	Confirmed
7	hsa_circ_0010541	hsa-miR-6860	Unconfirmed	22	hsa_circ_0051748	hsa-miR-762	Confirmed
8	hsa_circ_0001946	hsa-miR-135a-5p	Confirmed	23	hsa_circ_0051748	hsa-miR-4763-3p	Confirmed
9	hsa_circ_0001955	hsa-miR-145-5p	Confirmed	24	hsa_circ_0075341	hsa-miR-149-5p	Confirmed
10	hsa_circ_0002142	hsa-miR-625-5p	Confirmed	25	hsa_circ_0079070	hsa-miR-4463	Confirmed
11	hsa_circ_0003855	hsa-miR-145-5p	Confirmed	26	hsa_circ_0082878	hsa-miR-6860	Confirmed
12	hsa_circ_0006916	hsa-miR-522-3p	Confirmed	27	hsa_circ_0082879	hsa-miR-3187-5p	Confirmed
13	hsa_circ_0007915	hsa-miR-106a-3p	Confirmed	28	hsa_circ_0082879	hsa-miR-612	Confirmed
14	hsa_circ_0010541	hsa-miR-6827-5p	Unconfirmed	29	hsa_circ_0092306	hsa-miR-197-3p	Confirmed
15	hsa_circ_0001727	hsa-miR-330-5p	Confirmed	30	hsa_circ_0123996	hsa-miR-149-5p	Confirmed

## Discussion

In this paper, we introduce iHofman, a novel method for predicting CMIs by integrating multiple feature-extraction strategies with weighted attention mechanisms. By leveraging the sequence information from FastText, the structural context from GraRep, and the complementary representations of deep features from SAE, iHofman aims to capture the local and global features of CMIs. First, we use FastText to learn the sequence information representations of circRNA and miRNA, while GraRep captures the structural information representations of CMIs. To more effectively learn the deep information representations of sequences, we use SAE to extract high-order and low-order features. We then employ the weighted attention mechanisms to fuse the high-order and low-order sequence information representations with structural information representations. Finally, the fusion feature matrix is fed into a classifier to predict potential interactions between circRNA and miRNA.

The results obtained by iHofman are able to predict CMIs that may have a significant impact on cellular processes such as gene regulation, cancer development, and cell differentiation. Initially considered mere splicing by-products, circRNAs are now acknowledged as regulators of gene expression. They influence diverse signaling pathways by adsorbing and sponging miRNAs, thereby directly influencing both physiological and pathological processes. Moreover, circRNA-miRNA interactions decrease the number of miRNAs targeting other mRNAs, indirectly regulating the expression of target genes. The biological relevance of the model is further supported by case studies highlighting the potential of CMIs in disease mechanisms and treatment strategies. By providing more accurate predictions of these interactions, iHofman offers entirely new avenues to identify new biomarkers and therapeutic targets, particularly crucial in diseases like cancer.

In addition, we further investigated the nature of miRNAs identified by our model to assess their potential role as primary driver genes or minor passenger genes generated by pathological processes. For this purpose, we integrated existing literature with functional assessments. miRNAs predicted by iHofman have known roles in modulating oncogenic and tumor suppressor pathways, indicating their potential as drivers in certain disease contexts. For instance, miR-30 d and miR-34a are known to modulate critical signaling pathways in cardiovascular remodeling and inflammation [53]. This evidence implies that these miRNAs not only reflect pathological changes but potentially drive disease-associated phenotypes. Nonetheless, it is important to recognize that not all predicted miRNAs necessarily exert a causal influence. Some may function as passenger genes, influenced by upstream dysregulation.

The iHofman model presented in this study delivers solid performance, consistently achieving high scores across multiple evaluation metrics on all three benchmark datasets. The factors contributing to the effective performance of iHofman are as follows: First, FastText was utilized to extract representations from sequence information, while GraRep was employed to extract representations from CMIs network structure information. These representations were then combined to form a richer, more comprehensive representation. Second, SAE feature extraction enables the model to capture hierarchical data representations, effectively capturing both high-order and low-order features. Finally, the weighted attention mechanisms further enhance the model by focusing on the most relevant features, allowing the model to focus on critical interaction motifs and yielding deeper insights into the underlying dynamics of CMI formation.

Although the iHofman model shows strong promise in detecting CMIs, it is held back by two key limitations. First, its stacked autoencoder architecture can incur information loss during the encoding and decoding phases, potentially degrading overall performance and introducing uncertainty. Second, the current model fails to fully exploit complex interactions among input features, which restricts its predictive capability. Based on these issues, we aim to develop a more lightweight network architecture to reduce computational complexity and resource requirements while enhancing model performance.

## Conclusions

Extensive experiments across three benchmark datasets and one other domain dataset have shown that iHofman consistently outperforms the most advanced predictors. Notably, 26 of the top 30 novel interaction predictions were confirmed by independent biological databases, underscoring the method’s practical utility. These results validate iHofman as a powerful and reliable approach for uncovering potential CMIs. The integration of multiple feature extraction strategies with weighted attention mechanisms provides a comprehensive framework for capturing both local and global features of circRNA-miRNA interactions, contributing to the field of computational biology and offering new perspectives for disease biomarker discovery and therapeutic target identification.

Future research will address the current limitations of iHofman through the development of lightweight network architectures that preserve information integrity while managing complex feature interactions. We intend to expand the model application to additional RNA interaction types and investigate its utility in personalized medicine through integration with patient-specific genomic data. The promising results achieved thus far establish iHofman as a valuable instrument for enhancing our comprehension of regulatory networks between circRNA and miRNA, and their impact on human health and disease.

## Supplementary Information

Additional file 1. Table S1. The outcomes of the fivefold CV conducted on CMI-9589. Table S2. The outcomes of the fivefold CV conducted on CMI-20208. Table S3. The outcomes of the fivefold CV conducted on lncRNA-miRNA interactions dataset

## Data Availability

All data generated or analysed during this study are included in this published article, its supplementary information files and publicly available repositories: Zenodo (https://zenodo.org/records/15470088) [54] and GitHub (https://github.com/look0012/iHofman) [55].

## Abbreviations

CircRNA: Circular RNA
MiRNA: MicroRNA
CMI: CircRNA-miRNA interaction
SAE: Stacked autoencoder
AE: Autoencoder
MLP: Multi-layer perceptron
CV: Cross-validation
AUC: Area under the curve
AUPR: Area under the precision-recall curve
Acc: Accuracy
Sen: Sensitivity
Spe: Specificity
Pre: Precision
MCC: Matthews correlation coefficient
CNN: Convolutional neural network
DNN: Deep neural network
LSTM: Long short-term memory network
RF: Random forest
LR: Logistic regression
SVM: Support vector machine
KNN: *K*-Nearest neighbor
AdaB: AdaBoost
GB: Gradient boosting
