# Supplementary material for: iHofman: a predictive model integrating high-order and low-order features with weighted attention mechanisms for circRNA-miRNA interactions
Source: BMC Biol. 2025 Jun 9;23:162. doi: 10.1186/s12915-025-02260-5 (PMC12147305; doi:10.1186/s12915-025-02260-5)
Supplement: Supplementary file 1 — Additional file 1. Table S1. The outcomes of the fivefold CV conducted on CMI-9589. Table S2. The outcomes of the fivefold CV conducted on CMI-20208. Table S3. The outcomes of the fivefold CV conducted on lncRNA-miRNA interactions dataset [file 12915_2025_2260_MOESM1_ESM.docx]

**Additional file 1 for “iHofman: A Predictive Model Integrating High-order and Low-order Features with Weighted Attention Mechanisms for CircRNA-miRNA Interactions”**

Chang-Qing Yu^1,#^, Chen Jiang^1,#^, Lei Wang^2,3,*^, Zhu-Hong You^4,*^, Xin-Fei Wang^5^, Meng-Meng Wei^3^, Tai-Long Shi^1^ and Si-Zhe Liang^1^

^1^ School of Information Engineering, Xijing Univerity, Xi’an 710123, China

^2^ Guangxi Key Lab of Human-Machine Interaction and Intelligent Decision, Guangxi Academy of Science, Nanning 530007, China

^3^ School of Computer Science and Technology, China University of Mining and Technology, Xuzhou 221116, China

^4^ School of Computer Science, Northwestern Polytechnical University, Xi’an 710129, China

^5^ College of Computer Science and Technology, Jilin University, Changchun 130012, China

Supplementary Note: "The performance of iHofman on more datasets"

In order to demonstrate the performance of iHofman on different datasets, we utilized the widely used datasets in the circRNA-miRNA interaction prediction field, namely CMI-9589 and CMI-20208. CMI-9589, sourced from the circBank database, comprises 9,589 interaction pairs involving 2,115 circRNAs and 821 miRNAs. CMI-20208 contains 20,208 high-confidence pairs, covering 3,569 circRNAs and 1,152 miRNAs. Additionally, for external validation, we also conducted experiments on the lncRNA-miRNA interaction dataset. This dataset comes from the lncRNASNP2 database. After screening, this dataset contains 4966 experimentally verified lncRNA-miRNA interaction pairs. These interactions involve 770 unique lncRNAs and 275 miRNAs with available sequence information. We retrained and evaluated the iHofman model using five-fold cross-validation with the above three datasets. To conduct a strict evaluation of its generalization performance, we compared iHofman with the benchmark algorithms in their respective domains on different datasets.

For the CMI-9589 dataset, the results obtained by the model are presented in Table S1. Our model achieved an average accuracy of 83.76% with a standard deviation of only 0.75% across 5-fold cross-validation, indicating stable performance. The sensitivity reached 73.63% with a standard deviation of 2.89%, while specificity was notably high at 93.89% with a standard deviation of 1.42%. The precision value of 92.40% with a standard deviation of 1.37% further supports the reliability of positive predictions. The MCC value of 69.00% with a standard deviation of 0.93% confirms the overall strong predictive performance. The area under ROC curve and area under precision-recall curve values of 0.9187 and 0.9269, respectively, with very low standard deviations of 0.0029 and 0.0031, demonstrate excellent discrimination capability.

**Table S1** The outcomes of the 5-fold CV conducted on CMI-9589.

| 5-Fold | Acc.(%) | Sen.(%) | Spe.(%) | Pre.(%) | MCC.(%) | AUC | AUPR |
| --- | --- | --- | --- | --- | --- | --- | --- |
| 1 | 83.37 | 71.48 | 95.26 | 93.78 | 68.71 | 0.9216 | 0.9279 |
| 2 | 82.95 | 70.75 | 95.15 | 93.59 | 67.96 | 0.9205 | 0.9315 |
| 3 | 83.37 | 72.47 | 94.26 | 92.67 | 68.38 | 0.9194 | 0.9263 |
| 4 | 84.54 | 76.75 | 92.34 | 90.92 | 69.94 | 0.9141 | 0.9232 |
| 5 | 84.57 | 76.69 | 92.44 | 91.03 | 70.01 | 0.9181 | 0.9257 |
| Average | **83.76** | **73.63** | **93.89** | **92.40** | **69.00** | **0.9187** | **0.9269** |
| SD | 0.75 | 2.89 | 1.42 | 1.37 | 0.93 | 0.0029 | 0.0031 |

For the CMI-20208 dataset, the results obtained by the model are presented in Table S2. The experiments on the CMI-20208 dataset yielded comparable results, with an average accuracy of 83.92% and a standard deviation of 0.79%. Sensitivity was 74.95% with a standard deviation of 3.10%, and specificity was 92.89% with a standard deviation of 1.54%. The precision and MCC values were 91.40% with a standard deviation of 1.39% and 69.01% with a standard deviation of 1.02%, respectively. The AUC and AUPR values of 0.9173 and 0.9211 with minimal standard deviations of 0.0026 and 0.0020 further validate the model's generalization ability.

**Table S2** The outcomes of the 5-fold CV conducted on CMI-20208.

| 5-Fold | Acc.(%) | Sen.(%) | Spe.(%) | Pre.(%) | MCC.(%) | AUC | AUPR |
| --- | --- | --- | --- | --- | --- | --- | --- |
| 1 | 83.07 | 71.55 | 94.58 | 92.96 | 67.96 | 0.9172 | 0.9222 |
| 2 | 83.29 | 72.37 | 94.21 | 92.59 | 68.22 | 0.9187 | 0.9237 |
| 3 | 83.77 | 74.57 | 92.97 | 91.39 | 68.71 | 0.9130 | 0.9185 |
| 4 | 84.65 | 78.06 | 91.24 | 89.91 | 69.91 | 0.9198 | 0.9209 |
| 5 | 84.81 | 78.18 | 91.44 | 90.13 | 70.24 | 0.9181 | 0.9203 |
| Average | **83.92** | **74.95** | **92.89** | **91.40** | **69.01** | **0.9173** | **0.9211** |
| SD | 0.79 | 3.10 | 1.54 | 1.39 | 1.02 | 0.0026 | 0.0020 |

For the lncRNA-miRNA interactions dataset, the results obtained by the model are presented in Table S3. Our model maintained strong performance with an average accuracy of 82.90% and a standard deviation of 1.30%. The precision was 79.38% with a standard deviation of 3.05%, recall was 89.20% with a standard deviation of 2.42%, and F1-score was 83.93% with a standard deviation of 0.67%. The AUC and AUPR values were 0.9149 and 0.9214, respectively, with standard deviations of 0.0037 and 0.0065.

**Table S3** The outcomes of the 5-fold CV conducted on lncRNA-miRNA interactions dataset.

| 5-Fold | Acc.(%) | Pre.(%) | Rec.(%) | F1(%) | AUC | AUPR |
| --- | --- | --- | --- | --- | --- | --- |
| 1 | 82.91 | 78.75 | 90.14 | 84.06 | 0.9139 | 0.9256 |
| 2 | 81.05 | 75.24 | 92.58 | 83.01 | 0.9201 | 0.9285 |
| 3 | 82.42 | 78.52 | 89.26 | 83.55 | 0.9122 | 0.9150 |
| 4 | 84.52 | 83.44 | 86.13 | 84.77 | 0.9112 | 0.9142 |
| 5 | 83.59 | 80.94 | 87.89 | 84.27 | 0.9173 | 0.9238 |
| Average | **82.90** | **79.38** | **89.20** | **83.93** | **0.9149** | **0.9214** |
| SD | 1.30 | 3.05 | 2.42 | 0.67 | 0.0037 | 0.0065 |

The consistent performance across three datasets with distinct characteristics demonstrates the robust generalization capability of our model. The minimal standard deviations observed across all performance metrics indicate high stability and reliability. Notably, both AUC and AUPR values exceed 0.9100 across all datasets, confirming strong discriminative ability.
